# A Report on Some of the Cutaneous Diseases

**Published:** 1874-08-01

**Authors:** Hiram Nance

**Affiliations:** Kewanee, Illinois


### A REPORT ON SOME OF THE CUTANEOUS DISEASES.

READ BEFORE THE MILITARY TRACT MEDICAL ASSOCIATION, JULY 14, 1874.
BY HIRAM NANCE, M. D., KEWANEE, ILLINOIS.

MR. PRESIDENT AND GENTLEMEN OF THE MILITARY TRACT MEDICAL ASSOCIATION: To give you a full report on this branch of Practical Medicine would take a volume, and more than occupy the whole time allotted to the short sessions of our Society. And, consequently, I find it useless, and shall briefly call your attention at present to one class of diseases that have been sadly neglected by our profession. I allude to *diseases* of the *skin.* In fact, there is no class of diseases in the whole catalogue of medicine that is studied with less interest, and more abhorred by the student, with the exception of diseases of the eye, than cutaneous diseases. It seems that dermatologists, and teachers of ophthalmic medicine, in the embryonic stage of medicine, managed to place before their students the most perplexing and difficult nomenclature that could possibly be studied out; and this nomenclature, unlike the beautiful system of naming in chemistry, has been handed down to us moderns to puzzle our wearied brains over. Where is the medical student that can easily remember the diagnosis, or even the general symptoms of eczema, erythema, ecthyma and entozoa? And let me say to you (with humility) that there are few general practitioners that can readily give you the diagnosis between porrigo, impetigo, and psoriasis, or even prurigo, and scabies. And yet some of those belong to the class *squamous*, and some to the *pustular*, and one to the *animalcular*.

Some of our recent dermatologists, including Wilson, Neligan, etc., have done much to simplify our science by handing us plates of almost every skin disease, colored, and in atlas form; but this beautiful design is far below reality, and the practitioner, with the real disease and the photograph before his eyes, could hardly tell they were meant for the same. With these facts before us, shall we continue groping our way, not being able to diagnose, nor consequently treat our patient successfully? There is no class of diseases that will more readily bring reproach upon our beloved profession than the one we are now considering; for the trouble is not hidden as in ordinary diseases, but is plain to the sense of sight and feeling, and any unlearned person can easily see whether there is an improvement or not. The empyric rarely advertises that he is alone treating cutaneous affections. He prefers looking after more occult diseases that the public cannot so readily diagnose, by which he can deceive and draw his unearned money. Now, gentlemen, if you want to maintain the integrity and high standing of our profession, I would call you especially to look after this branch of medicine. I have felt my incompetency often in treating skin diseases; not that I did not diagnose my cases usually correctly, but that when I did, I found that our remedies are often useless, and even sometimes aggravating. What will one time cure psoriasis, another time will utterly fail; what will at one time remove every vestige of porrigo, at another time seems to give life and vigor to the disease. With these assertions and facts before our eyes, look well to your cutaneous patients before giving a prognosis, and then give it very gradually. Some of those cutaneous diseases are really incurable so far as our science at present extends; but there are but few of them but what can be much ameliorated by a wise and judicious treatment. If we are thoroughly versed we can readily give our opinion to our patient, and thereby save him from much anxiety; for should we give a prognosis that he would be well in three months, and the disease continue six months or a year, it would not be likely that he would in future place much confidence in our medical judgment, and that our services would be entirely dispensed with. We humble ourselves and the profession enough by saying that the disease is stubborn and unyielding, and it may take weeks, months or years, and may be entirely incurable. But if we diagnose correctly, we can give just such an opinion, and it will prove in the end correct.

I beg to briefly call your attention to a few of the most common of this class of diseases. And first I will mention *pityriasis*. This is one of the *squamæ*, and is characterized by an abundant secretion of minute white shining scales, the patches occurring on all parts of the body and limbs, and varying in color from dark brown to a yellowish hue. Some authors divide it into three or four varieties, calling it *pityriasis rubra*, *pityriasis nigra*, and *pityriasis versicolor;* but this division can be easily dispensed with, and the division brought down to *pityriasis diffusa* or general pityriasis, and *local pityriasis* or *pityriasis capitis*. Wherever found this disease always has its peculiar diagnostic marks, and the dermatologist need not have much difficulty in his diagnosis. It is characterized by itching and tingling; is not contagious; so you rarely see more than one patient in a family suffering from it. The constitution rarely suffers from it, although it is wont to run a very chronic course. It may occur on any part of the body, but I have found it as general on the legs and arms as on the chest, or all around the thorax. The spots usually come out with a reddish appearance, with a burning tingling sensation, and in a few days fade to a yellow or dark stain, and then accompanied with its peculiar small scales. These continue from day to day, from week to week, and sometimes from year to year, being almost entirely unyielding to treatment. The above description is as you usually find it on the surface of the bare skin uncovered by hair. It is diagnosed from psoriasis by the fineness of the scales, and by the spots usually not being so large, and by its usually spreading so much more rapidly; by the great change in the color of the skin, and by its being rarely elevated above the surface, and also by its almost continued pruritus. It need not be confounded with eczema, nor ichthyosis; for any medical man giving skin diseases any attention at all could easily tell the difference. *Pityriasis capitis* might be more difficult to diagnose, for we frequently find it existing on the scalp, when there is not a vestige of it on any other part of the limbs or surface of the body. When it occurs on the scalp you will not find a sign of ill health. The patient's attention is called to the disease from its continued itching, unattended with any heat, burning, or redness of surface, and on examination we find a secretion of furfurraceous scales, called in common language *dandruff*. This itching with many persons is almost intolerable; the hair sometimes comes out and leaves the surface bare, and the hair becomes dry and crisp, and Dr. Neligan says baldness may result, but this is only temporary, except in old persons. I now have two patients under my care, one with general pityriasis, and the other from pityriasis capitis—the former only recently under treatment, and is on pil. hydrarg. and saline purgatives once every three or four days, and from twelve to fifteen drops of Donovan's solution. Locally I am using a wash of one-half drachm of carb. potass. to one pint of water every morning, and when dry, sulphur ointment on all the patches. As the disease involved the scalp, as well as the general surface, I had the hair cut short and the same applications made to it. The treatment has not been continued long enough to show the result. The other case has been under treatment for several months, and is entirely confined to the scalp; patient perfectly well every other way. I have only used local applications, and every remedy I use seems to relieve, but none seem to cure. She has been directed to use bichloride of mercury in a solution of from three to five grains to the ounce, carb. potass. wash, and various ointments containing calomel, camphor, glycerine, etc. Should the case continue I will place her on the arsenic treatment, and also sulphur or lead ointments, or, as Dr. Fraser recommends, a weak solution of tannin in glycerine. Last winter, I had a case in a man aged about forty-eight. I placed him on Donovan's solution and used as a local application aqua rosa combined with chloroform. This acted like a charm, and I only had to renew the treatment once until my patient was cured. While this case yielded so readily, my other cases had not been so fortunate.

Your attention is called, secondly, to another disease, classified among the *squamæ*, and of equally frequent occurrence as pityriasis, which is as difficult of treatment and perfect cure. I refer to *psoriasis*. The outlines and general appearance of psoriasis are so well marked that no physician making any pretense to the understanding of cutaneous diseases need err in a diagnosis. There is but one disease that the uneducated physician or student would at all be likely to confound with it, and that is the one just passed over. Psoriasis in common language is called dry tetter—*dry scale*. And many women will call to see you and say they have *salt rheum* on their hands and fingers; this latter is only a variety of psoriasis, but yet it comes under that head. As most commonly seen on the limbs and body you will find irregular spots, varying in size and shape, and covered with scales of a bright and shining appearance, and about the size of a bean. The spots are depressed in the centre, but elevated at the edges. But as the disease advances these spots coalesce and become confluent, and run into one mass of scales, and then the usual redness that is seen in its formative stage disappears. We now have patches varying in size of a dollar to a whole surface of one or both legs. Or, instead of a limb being occupied by it, you may find a patch of the size of the palm of the hand on the shoulder, another on the arm, and so scattered all over the cutaneous surface. The diagnosis of psoriasis, as I before remarked, is comparatively easy, for the surface is covered with scales, and it is rare, if ever, you find any oozing of a serous or sero-purulent discharge as you find in eczema, herpes, or lichen, and there is not that itching and burning that you find in any of those diseases. It stands prominent as a squamous or scaly disease, and I cannot fix your minds more permanently upon it than by telling you that in my early practice I was called to see a man, aged about forty or forty-five, who informed me he was suffering under an incurable disease, and had been treated by a number of English physicians, none of whom had benefited him in the least. The disease was principally upon his legs—and before exhibiting them to me, I must tell you he was in his parlor, but in this parlor his young stock of poultry were not excluded, but were commoners over the whole house. On rolling up his pants and gently rubbing the surface the scales fell like flakes of fine snow. The chickens seeing them, ran for the coveted prize, and devoured them as if they were the most choice morsels. This case was immediately placed on saline laxatives; good diet; Fowler's solution in ten-drop doses three times a day, the surface to be well cleansed and the *ung. picis liquidæ* applied every night. This treatment acted charmingly, and I had the satisfaction of believing my patient cured. I knew him several years after and never heard of a relapse. As a general rule I think this treatment is a good one, and if sufficiently persisted in, will wholly effect a permanent cure. But when psoriasis occurs alone on the palms of the hands, on the fingers, on the nails, and between the fingers, it seems to be an entire different disease, and I don't know the correct classification; for if we are fortunate enough to cure it on the hands it is almost certain to return the next winter. Sometimes I cure this *psoriasis palmaris* with an ointment of ammoniated mercury; sometimes with ung. hydrarg. nit.—tinct. iodine—and I have found one drachm of sul. acid to one or two ounces of water occasionally applied very beneficial. The hands should not be placed in water often. One case confined entirely to the fingers I removed entirely by bandaging and applying cold water; the treatment was continued several weeks, but I think there was no relapse.

My report must necessarily be brief. So I leave *psoriasis* and call your attention to *sycosis* or *mentagra*, so called from its being wholly on the chin. It is a disease of a parasitic vegetable production, always occurring on the parts of the face where the beard grows, and seeming to commence at the roots of the beard with round papular elevations, with a dry grayish scurf. This continues until scabs form; the surface becomes rough, uneven, and the appearance becomes hideous; the scabs loosen; then we have blood and sanious pus, and when the parts heal it leaves reddish stains; when these scabs have loosened, in a brief time the surface again becomes rough, and a repetition of maturation and decline is the result; and so the disease, without proper remedial measures, will continue from month to month and from year to year. *Sycosis* is decidedly a contagious disease, but fortunately women are exempt, for they are not troubled with that hirsute appendage so much admired by them in the opposite sex. I think nearly, if not quite all cases, are produced by persons frequenting barber shops, and being shaved by razors having been used on persons contaminated with this loathsome and horrid malady. The *diagnosis* is not difficult. I cannot see how any intelligent physician can err if he gives the necessary attention to his patient. He may mistake it for syphilitic eruptions, but these he will not find confined to the chin and parts covered with beard. And there is not the copper-color and elevated edges that are found in syphilitic eruptions. It certainly cannot be mistaken for empetigo or ecthyma, and even should it be, the treatment would not materially differ. But a grave error would be committed in the former case, for syphilitic troubles should have the prompt advantage of mercurials. I have rarely found much difficulty in treating *sycosis*. The patient is usually suffering under some derangement of the digestive organs. And in one or two instances in my practice young men have came to me, having a hypochondriac appearance, seeming as if they were on the verge of mental derangement. In such cases I always find torpidity of the liver and other chylopoetic viscera, strongly demanding a combination of mild mercurials, pulv. rhei., and the usual vegetable tonics. The bowels should be moved once or twice a day by such laxatives, and the tonic given at least three times a day before every meal. Under this treatment, with a judicious regimen, you will find your patient's general health very much improved in a week or ten days, and then I place him on Donovan's solution, in from ten to eighteen drops after every meal, and thus continue him for months if necessary. I have rarely found it necessary to use a variety of local applications. The one that I usually prefer is an ointment composed of simple cerate, calomel, acetate of lead, and chloroform. This should be applied once or twice a day, and before making the application the chin and face should be gently sponged over with milk and water, or a mild solution of acetate of lead. The razor should not be allowed to touch the face during the treatment, for it would aggravate the healing process which may be established by our remedies, and all soaps and irritating cosmetics should be sedulously avoided. How very necessary it is to understand the pathology and treatment of such a repulsive disease in its infancy; for we are told that sometimes it becomes irremediable. And I cannot but feel happy, that with all the repulsiveness, hideousness, pain and general distress that our patients suffer under, we have remedies that, if judiciously applied, will remove every trace of this loathsome malady. As *sycoris* is very liable to return after every vestige of the disease is removed, we should not relax our treatment too hastily, but continue the treatment for a month or two after it has apparently all been eradicated from the system.

My duty is not done in this case until I urge upon every member of our Society the importance of guarding the inexperienced who frequent the barber's shop not to be shaved with contaminated razors. And the the barber who thus inoculates his patron should be held responsible for all the suffering brought upon him. When sycosis has been permitted to continue for a great length of time the patient loses his beard in spots, never to be regained; and these lost patches are also partially changed in color, giving the countenance a very peculiar and homely appearance.

Having given you the outlines of *psoriasis* and *sycosis*, I now hastily direct your attention to a disease that is of not unfrequent occurrence. I refer you to *porrigo*. *Porrigo*, also called in common language *scald-head*, and by the profession *tinea capitis*, is one of the grave cutaneous diseases, for its duration is of indefinite time. Probably no disease affecting the cutaneous surface causes more alarm among the mothers of the affected children than *porrigo*. Tell the mother that her child has *scald-head*, and she is frantic; for the public have learned just enough to know that it is stubborn to our remedies, if not in some instances incurable. But, gentlemen, I take no such unfavorable view, but claim that nearly every case is amenable to a very simple treatment if persistently applied. Porrigo is easily diagnosed, but let me say that it is not entirely confined to the hairy scalp, but you not unfrequently find it making its appearance on the neck, and down on the back. It is usually developed in small elevated dry spots, which grow larger and larger, and coalescing, run into a crust, and then into irregular masses, spreading all over the scalp, forehead, down the neck, and sometimes down over the trunk, the whole parts being covered in one honey-comb-like yellow mass of disease. The color is yellowish, and the scabs hard and dry; they break up in a meal-like substance, but when removed they usually bleed, and leave a sore surface beneath. The hair becomes diseased, short and crisp; much of it falls out, leaving a straggling one here and there; bald spots are not unfrequent, and this baldness usually remains permanent. It is truly pitiable to look upon the disgusting little object before us when in this condition; but when we feel a confidence that remedies, if properly applied, will cure, then it is we take courage, and feel that our mission as members of the healing art is of much value. Porrigo, I said, was not difficult of diagnosis, but it may be mistaken for *herpes capitis*, but this would not be a serious error, for the same treatment would cure either one. I have been in the habit of treating porrigo with two or three kinds of external remedies, and always, if persistently continued, have I been richly rewarded with a cure. My internal remedies have always been the same. Locally, I order the hair to be cut as short as it possibly can be, and kept in this condition throughout the long continued treatment. If the head is covered with crusts, order linseed meal poultice to be kept on during the the night. Next morning remove the poultice and wash the head with a solution of carb. pot., one teaspoonful to one pint of soft water; then apply freely *ung. picis liquidæ*, and continue this treatment every day or every other day for several months. If upon thorough trial I find my treatment not succeeding, I change and wash the head with castile soap and soft water every third day; then apply a solution of twenty or thirty grains to the ounce of nitrate of silver. Either of these local treatments, if persistently carried out, will reward you with a cure. I omitted to say, that after the application of the silver solution, when the surface becomes dry, that I have the nurse apply the *ung. hydrarg. nitratis.*

Porrigo, when of long duration, leads to serious ill health, and I have frequently seen my little patients come to me with a vacant, idiotic stare, showing that the brain and general nervous system was seriously affected, leading to a state of almost, if not quite, dementia. This being the true state of the system, a constitutional treatment is imperatively demanded, and I place my patient on mild alteratives, tonics, etc., to invigorate the system, and, amongst these remedies, I know of none better than iodide of arsenic in from one-tenth to one-twelfth grain doses to a child ten or twelve years old, and proportionately less for younger persons. Or, instead of the iodide of arsenic, I believe Donovan's solution in drops from four to ten is of equal value. Should the bowels become torpid give hydrarg. cumcreta with small doses of pulverized rheum and magnesia.

Porrigo is of a *mycelium* or *vegetable fungus* origin, and only wants the right kind of soil to develop itself in and grow prolifically; and if any of the particles of fungi are transmitted to the right soil, contagion by inoculation is the result. Young people, especially children under ten or twelve years of age, are the persons usually affected, but older children, and even adults, have been known to contract the disease. The most assiduous care should be taken in schools, and places where children congregate, to prevent any source of contagion. Children should never use the same comb, towel, wash-basin, bonnet, hat, or any other thing worn by the diseased, for infection is almost sure to follow. Neither should children of the same household be permitted to sleep in the same bed or lounge, for the *fungi* or *mycelium* left on the pillow would readily communicate the disease to the ones thus exposed.

For fear of making my paper too long, I lastly call your attention to *herpes*. Herpetic eruptions occur so frequently and under such a variety of forms that it requires quite a dermatologist to keep trace of them—so named by the ancients from *serpo, I creep*. The general features of herpes, no matter where occurring, have certain outlines that can easily be distinguished, with the exception of *herpes capitis*. This latter bears such a close resemblance to *porrigo scutulata*, that I must say few physicians would commit much of an error in pronouncing the disease the same; yet the circular-like form of herpes capitis would be a good diagnostic mark. You will diagnose this disease by its making its appearance in from twelve to twenty-four hours from the time the first symptoms make their appearance in the cutaneous surface; patient feels a burning and tingling sensation in the parts to become the foci of irritation; the surface becomes red and inflamed, and then on this red surface the eruption of minute vesicles appear, and rapidly grow to the size of *bullæ*, especially in the variety called *herpes zoster*. *Herpes* occurs in so many places on the surface that it has received the names of the particular locality — thus *herpes labialis*, herpes preputialis, herpes capitis, etc. But for practical purposes it is enough to divide it into three varieties, viz.: H. phlyctenodes, H. zoster and H. circinatus. The first variety, *herpes phlyctenodes*, may occur on any part of the cutaneous surface, and usually, in twenty-four hours from the time of the burning and tingling sensation, the eruption makes its appearance, and the vesicles on the inflamed surface rapidly grow and sometimes become as large as a small pea, though the majority of them usually remain small; the patches vary in size from a shilling or half-dollar to patches the size of the palm of the hand; in three or four days the vesicles break, having a sero-purulent matter in them; these form crusts and scabs, which fall off, and a new set again appears to run through the same stage. In these cases the system is usually deranged, loss of appetite, foul tongue, aching of the bones, etc. I give a dose or two of hydrarg. chlorid. mite or comp. c. pills, with some vegetable tonic, and paint over the diseased spots tinct. iodine, to be followed by ung. hydrarg. nitratis. This treatment alone in this variety will usually effect a cure in eight or ten days. For herpes occurring on the lips, wash with a solution of sul. zinc in rose water, and apply ung. calaminæ. For herpes preputialis, keep the parts thoroughly cleansed and wash with black or yellow wash. And be sure your patient hasn't got *chancre*, for this would be a serious error. The diagnosis is plain, and any learned physician should be able to make it.

The next variety is *herpes circinatus* or ring worm. The diagnosis is easy, and the mild variety is usually of not more than twelve or fourteen days' duration; it mostly occurs on the neck, cheeks or face, but may occur any place. For years I have treated this variety with a saturated solution of sodæ boras in diluted acetic acid. This will remove it in nearly every instance, without any constitutional treatment. When it fails, tinct. iodine is usually all that is required.

The variety (if such it be) called herpes capitis, so much resembles porrigo that they are frequently blended and treated for the same disease. They are distinguished by the former being more circular, and the crusts not being so hard and friable, but both are stubborn and unyielding to treatment; but I have always found them to yield finally under the treatment laid down under the head of *porrigo scutulata*.

The last general variety I shall notice is *herpes zoster* or *shingles*. This type of the disease is supposed to originate from some perversion or lesion of the nervous system. It always occurs on the side of the thorax or abdomen, and occasionally you will find groups of it on the neck, and rarely ever down on the thigh. It comes on, as other varieties of this disease, with symptoms usually more aggravated—heat, burning, pricking sensation, with redness where the spots are going to appear; and in twenty-four hours or less you will find groups or patches appearing distinct from each other, vesicular in form, rapidly running into *bullæ*, frequently many of them as large as a small pea, much resembling the appearance of small vesicles produced by emp. cantharidis before the blister is perfectly formed. It is astonishing, sometimes, the amount of constitutional disturbance the formative stage of this disturbance will set up. We not unfrequently have chills, fever, nausea, vomiting, biliary derangement, etc., with severe neuralgic pain, connected with burning, loss of sleep, etc. And even after the external disease is entirely removed, the severe burning and pain, with some persons, will continue for months, and sometimes years. This shows conclusively and certainly that the sentient portion of the nerves is seriously implicated. It is useless to spend time in describing this disease more minutely. Some eighteen or twenty years ago, when collodion was first discovered and brought prominently before the profession, it occurred to me that this was just the remedy for *shingles*, and I resolved the first case I had to give it a trial. Very soon the case came, and I used it *plentifully* and *successfully*, and with entire satisfaction. In fact, I must say it acted *charmingly*. Every case since that time has been thus treated by me, and always with success. If there is a specific in any disease, it is certainly collodion painted all over the eruption once or twice a day. I have never known it to fail in checking it and drying up the eruption. Sometimes it is necessary to heal up the ulceration left after the drying up of the *vesicles* and *bullæ* by a weak lead wash, and *ung. calaminæ*. Of course a mild alterative treatment is certainly demanded. I was so pleased with the treatment at the time, that I wrote an article for one of the Chicago Medical journals, and it was duly published; and I am now, gentlemen, happy to reiterate what I then wrote, and thereby corroborate my former experience.

Finally, let me say that no medical college could do a better thing for the general public, for their alumni, and pecuniarily for themselves, than to have wax casts of all cutaneous diseases in their museums, and a special chair established on dermatology. All the efforts made by Wilson, Neligan, and a host of others to have plates engraved to correspond with the real disease have sadly failed; but wax casts are immediately recognized. On visiting the museum of the New Orleans Medical College a few years ago, and seeing a cast of sycosis, I was perfectly struck with the *fac simile;* and any medical student would readily read the real from the cast.

